# The effect of epidural education on Primigravid Women’s decision to request epidural analgesia: a cross-sectional study

**DOI:** 10.1186/s12884-018-1766-5

**Published:** 2018-05-03

**Authors:** Maha Heshaam Alakeely, Arwa khalaf Almutari, Ghadah Abdulrhman Alhekail, Zainah Ahmad Abuoliat, Alaa Althubaiti, Laila Abdul-Rahman AboItai, Hanan Al-Kadri

**Affiliations:** 1 College of Medicine, King Abdullah International Medical Research Centre /King Saud bin Abdul-Aziz University for Health Sciences, NGHA , Riyadh, Saudi Arabia; 2 Department of Basic Medical Sciences, College of Medicine, King Saud bin Abdulaziz University for Health Sciences/King Abdullah International Medical Research Centre, Riyadh, Saudi Arabia; 3 Department of Health Education, King Abdullah International Medical Research Centre /King Saud bin Abdul-Aziz University for Health Sciences, NGHA, Riyadh, Saudi Arabia; 4 Department of OB/GYN, King Abdulaziz Medical City, College of Medicine, King Saud Bin Abdulaziz University for Health Sciences/King Abdullah International Medical Research Centre , Riyadh, Saudi Arabia

**Keywords:** Primigravid women, Epidural analgesia, Education

## Abstract

**Background:**

Epidural analgesia represents one of the most effective pharmacological ways to relieve labour pain. Women’s awareness regarding the use of epidurals is increasing. As the decision to use epidural analgesia during labour is affected by many social, personal and medical factors, this study aimed to explore the factors contributing to a pregnant women’s decision to use epidurals and to understand the benefit of implementing a health education program regarding epidural analgesia.

**Methods:**

A cross-sectional study was conducted with primigravid women visiting the Obstetric Clinics for their routine antenatal care at King Abdul-Aziz Medical City in Riyadh from October 2014 to December 2016. The participating pregnant women were educated on the use of epidural analgesia during labour by a professional health educator utilizing specially designed educational materials. We assessed the relationship between the women’s decision to request epidural analgesia and their age, place of residence, occupation, income and education level using a questionnaire.

**Results:**

A total of 81 primigravid women were included in the study. Employed pregnant women were more likely to request epidural analgesia than non-employed women (46.7% vs. 18.2%, *P* = 0.019). After education, significantly more pregnant women were planning to request epidurals (mean score for answers before education was 2.12 ± 0.578 vs. 2.27 ± 0.592 after education, *P* = 0.013). Other variables, such as age, level of education, income and place of residence were not significantly associated with the participants’ decision to request epidural analgesia.

**Conclusion:**

Health education on epidural analgesia is an important factor in increasing primigravid women’s desire to request epidural analgesia. Education on epidural analgesia during antenatal care is needed for better decision making regarding the use of epidural analgesia during labour.

**Electronic supplementary material:**

The online version of this article (10.1186/s12884-018-1766-5) contains supplementary material, which is available to authorized users.

## Background

Childbirth is an important life event; however, the pain caused by labour is far more intense than pain from other causes. Canadian women’s scores regarding labour pain were higher than those for pain due to any other causes [1]. Epidural analgesia is an effective pharmacological technique used to manage labour pain. It involves the injection of an anesthetic into the lower back to decrease the sensation of pain felt during labour. Although epidural analgesia may increase the risk of instrumental delivery, it is not associated with increasing risk of caesarean section, causing chronic back pain or affecting the neonate’s Apgar score [2]. Women’s awareness regarding the use of epidural is increasing. Of the 4 million women who give birth each year in the United States, 1.6 million are likely to use epidural analgesia during labour. [3] Another study in Finland showed that 66.55% of women with first vaginal delivery of singleton pregnancies had requested epidural analgesia for labour pain relief [4]. In Brazil, women who requested epidural analgesia were satisfied with their experience and 97% of them would consider going through the same procedure at future deliveries [5]. The decision to use epidural analgesia during labour is affected by many factors such as the culture and background of the women, their knowledge, financial status, and educational leve [6]. Epidural analgesia in labor is practiced in most of the delivery centers in Saudi Arabia. The aim is to provide the delivering women with an effective method of pain relief in labor. At King Abdulaziz Medical City, the department of Obstetrics and Gynecology is conducting about 8000 deliveries every year, more than one fifth of these deliveries are cesarean deliveries. The department offers this service to all patients in labor who will accept the procedure and are in need for. The procedure is done by the on-call anesthesiologist in labor and delivery area after taking an informed consent. All anesthesia physicians that are assigned to labor and delivery are well trained on the procedure, follow strict internal policy and procedure on its performance and are monitored by an anesthesia consultant throughout the day. It was observed that there is variation in the women ‘acceptance to go for this type of labor analgesia. This variation is affected by several medical and social misconceptions as well as religious and spiritual factors. Scant studies were done on Saudi women perception of epidural analgesia in labor and to our knowledge no Saudi study was done on the effect of antenatal education on women perception and acceptance of the procedure in labor [7]. A previous study conducted in Saudi Arabia showed that higher income and greater knowledge of epidural analgesia positively affected their choice to use epidural analgesia during labour [6]. Moreover, the same study demonstrated that a higher level of education in women corresponded to an increase in their knowledge, which may positively affect their awareness of epidural analgesia as an available choice for relieving labour pain in the future. [6] According to a survey of Nigerian women, out of 650 women, only 127 were aware of epidural analgesia [8]. Therefore, during pregnancy, an important role of antenatal care services for pregnant women should be to educate them on analgesia and pain relief methods during labour. With this awareness, women can then decide whether to consider these options during labour. Since there is misconception about epidural analgesia in the society and lack of research about this topic in Saudi Arabia, the aim of this study was to evaluate the knowledge of primigravid women regarding the indications and side effects of epidural analgesia and their sources of information in this process. Additionally, the study aimed to explore primigravid women’s awareness of epidural analgesia and the factors affecting their decisions to request epidural analgesia at the National Guard Health Affairs/King Abdul-Aziz Medical City in Riyadh. Moreover, we determined the effectiveness of an epidural analgesia educational program.

## Method

### Study design, area and setting

This was a cross-sectional study using a self-administered questionnaire conducted from October 2014 to December 2016 in Obstetrics and Gynecology (OB/GYN) department, outpatient clinic 101, National Guard Health Affairs (NGHA)/King Abdul-Aziz Medical City in Riyadh, Saudi Arabia.

### Identification of study participants

The inclusion criteria were pregnancy, age between 19 and 35 years, Saudi nationality, primigravity, and never being participated in an educational session on epidural analgesia. Exclusion criteria were applied if participant had contraindication to the use of epidural analgesia such as spine abnormalities, coagulation abnormalities and plans to receive elective cesarean delivery.

### Data collection process

A convenience sampling technique was used. A trained health educator working at the NGHA hospital, OB/GYN department performed the data collection based on the inclusion and exclusion criteria. During visits for their routine antenatal care, primigravid women were invited individually to the health educator’s room in clinic 101. The health educator then explained the study to them. After they agreed to participate in the study, they were asked to sign an informed consent. The primigravid women completed a questionnaire that assessed their perceptions and awareness of epidural analgesia. The variables in the questionnaire were age, education level, income, occupation, source of previous knowledge of epidurals, and awareness of the usage of epidural analgesia, its indications and side effects (Additional file 1). After they had completed half of the questionnaire, the primigravid women were educated by the health educator about the use of epidural analgesia during labour using a Power Point presentation. Each session lasted between 15 to 30 min, and the participants had the opportunity to ask questions during the session. The target of the education is to simply educate women about options for pain relief.

After the education, the same questionnaire was distributed, and the primigravid women were asked to fill out the rest of the questions, which mainly included questions asking whether they would accept epidural analgesia during labour. The instrument used for data collection was a self-administered questionnaire distributed to 81women. The questionnaire was designed based on a literature review and translated into Arabic by the co-investigators of the study then judged by asking six primigravid women and 3 experts in the field, two obstetric gynecologists and a consultant anesthesiologist, about their opinions on the items. The questionnaire was then revised based on their remarks. The educational material included a set of PowerPoint slides that were displayed on a large TV screen in the health educator’s room. The slides included the definition of epidural analgesia, the procedure, and the advantages and disadvantages of epidural analgesia. The educational material was translated into Arabic by coinvestigators and judged by 3 experts in the field, i.e., two obstetric gynecologists and a consultant anesthesiologist, who provided their opinion on the educational material; the material was revised based on their remarks.

### Data analysis

The event rate of epidural analgesia is about 25% of all primigravid [7]. With a power of study 80%, level of precision 10%, and two-sided significant level 5%, the minimum sample size required is 73 subjects. Assuming 20% non-response or dropout rate, the final sample size is 91 subjects. The data were presented as the mean ±standard deviation for continuous variables and frequencies (percentages) for categorical variables. Chi-squared test or Fisher’s exact test was used to compare categorical data (such as culture, knowledge, income, education level and the decision to request epidural analgesia). A dependent sample t-test was used to compare women’s agreement to use epidural analgesia before and after the education, with higher scores indicating stronger agreement. Women who responded “no” or “not decided” were grouped into one category. A *p*-value<0.05 was considered statistically significant. The data were analyzed using the Statistical Package for the Social Sciences, Version 20.0 (IBM Corporation, Armonk, NY, USA).

## Results

### Demographic characteristics

Table 1 shows the demographic characteristics of the participants. A total of 81 primigravid women participated in this study and completed the questionnaire. The mean age of the pregnant women was 26 ± 4.10 years. Twenty-seven (33.3%) women had completed college-level education, 66 (81.0%) were unemployed, 42 (51%.9%) had a monthly income between 5000 and 10,000 SR, and 68 (84%) were urban residents.

**Table 1:** Demographic characteristics of the study participants (*N* = 81)

Characteristics	N (%)
Age, y	
< 26	40 (49.4)
> 26	41 (50.6)
Education	
Primary school	1 (1.2)
Middle school	4 (4.9)
High school	27 (33.3)
College	43 (53.1)
Postgraduate	6 (7.4)
Employment status	
Employed	15 (18.5)
Not employed	66 (81)
Income	
Less than 5000 SR*	25 (30.9)
5000 to 10,000 SR	42 (51.9)
10,000 to 15,000 SR	9 (11.1)
More than 15,000 SR	5 (6.2)
Place of residence	
Urban	68 (84)
Rural	13 (16)

*1 SAR = 0.266551 USD

### Assessment of the perceptions of primigravid women toward epidural analgesia before education

Table 2 describes the perceptions of primigravid women toward epidural analgesia. Out of the 81 primigravid women, 31 (38.3%) thought that epidural analgia was the most effective method of pain management. From those that knows about epidural analgesia, 19 (61.3%) considered pain management a major reason for requesting epidural analgesia, and 16 (51.6%) mentioned the possible risks of epidural analgesia (such as back pain and headache) as their main concern. Family members/friends were the main source of information in 18 (58.1%) women, and the opinion of the partners were neutral for 23 (74.2). Before receiving education on epidurals, 48 (59.3%) women were undecided about requesting epidural analgesia in the future, 14 (17.3%) said yes, and 19 (23.5%) said no.

**Table 2:** Perceptions of primigravid women toward epidural analgesia before education

What do you think is the most effective means of pain control during labour?	N (%)
No effective method	2 (2.5)
Pain relief with intramuscular analgesia	3 (3.7)
Pain relief with intravenous analgesia.	3 (3.7)
Pain relief using gas inhalation	2 (2.5)
Epidural analgesia	31 (38.3)
Other	10 (12.3)
Do not know	30 (37)
What was the number one reason you might have wanted an epidural for labour?	
Pain control	19 (61.3)
Relief of fatigue/stress	7 (22.6)
Encouraged to obtain epidural by friend/family member	2 (6.4)
Encouraged to obtain epidural by OB, midwife, labour educator	3 (9.7)
Other	0 (0)
What was the concern you had that may have led you towards avoiding an epidural for labour?	
Concern over possible risks to me (back pain, headache, etc.)	16 (51.6)
Concern over possible risks to baby	5 (16.1)
Pain from needle/procedure	4 (12.9)
Desire for natural childbirth	3 (9.7)
Concerns about me from the community	1 (3.2)
Other	2 (6.5)
What was your main source of information on epidurals prior to your labour?	
Physician	15 5(16.1)
Midwife	2 (6.5)
Family member/friend	18 (58.1)
Book/video/TV program	1 (3.2)
Childbirth class	1 (3.2)
The Internet	4 (12.9)
Did your partner prefer that you receive an epidural for labour?	5 (16.1)
Yes	3 (9.7)
No	23 (74.2)
Neutral	ᅟ
In the future, will you request an epidural when in labour?	
Yes	19 (23.5)
No	9 (11.1)
Not decided	53 (65.4)

### Association between demographic characteristics and epidural request before education

Table 3 presents the associations between demographic characteristics of the participants and epidural requests. Among the variables that we assessed, age, level of education, income and place of residence were not significantly associated with women’s desire to receive epidural analgesia (*P* = 0.798, 0.435, 0.939, and 0.281, respectively). Employed pregnant women were more likely to request epidural analgesia than non-employed women (46.7% vs. 18.2%, *P* = 0.019).

**Table 3:** Association between demographic characteristics and epidural request

Demographic characteristics		Yes N (%)	No/not decided N (%)	*P* value
Employment status	Employed	7 (46.7)	8 (53.3)	0.038
	Not employed	12 (18.2)	54 (81.8)	
Education	School level	9 (28.1)	23 (71.9)	0.435
	College and postgrad	10 (20.4)	39 (79.6)	
Age, y	< 26	10 (25.0)	30 (75.0)	0.798
	> 26	9 (22.0)	32 (78.0)	
Income	Less than 5000 SR	6 (24.0)	19 (76.0)	0.939
	5000 to 10,000 SR	7 (16.7)	35 (83.3)	
	10,000 to 15,000 SR	6 (42.9)	8 (57.1)	
Place of residence	Urban	18 (26.5)	50 (73.5)	0.281
	Rural	1 (7.7)	12 (92)	

Based on Chi-square or Fisher exact test

### Assessment of attitudes toward epidural analgesia after education

The results of the answers to the questions “In the future, will you request an epidural when in labour” vs. “After receiving the education about epidural, will you request an epidural when in labour” were significantly different. After education, more pregnant women displayed willingness to request an epidural. The mean score for the answers before education was 2.12 ± 0.578 vs. 2.27 ± 0.592 after education (*P* = 0.013). In addition, after education, more women agreed on the need to be educated about epidural analgesia before labour, 79 (97%). Out of all the educational methods mentioned (Table 4)), the majority of women (35, 44.3%) showed preference to be educated during doctor consultations.

**Table 4:** The preferred mean of education among the participants

Means of education about epidural analgesia	N (%)
By means of a pamphlet that I can read	2 (2.5)
By means of a video that I can watch	7 (8.9)
During antenatal talks by the nursing staff	24 (30.4)
During the doctors’ consultation	35 (44.3)
In a special session by the anesthetist	11 (13.9)

## Discussion

Our survey revealed that primigravid employees are more willing to request epidural analgesia compared with non-employees. In addition, education regarding epidural analgesia is important in predicting the primigravid desire to request epidural analgesia.

### Comparison with other studies

We assessed various factors that may affect pregnant women’s decisions to request epidurals during labour before they were educated on the matter. Among the factors, employment was a significant predictor of women’s requests for epidural analgesia during labour. Previous studies identified different factors as predictive of an epidural request. Educational level is a significant factor associated with epidural request [6–10]. Moreover, our findings were not consistent with the different factors that have been previously shown to be significant. Factors, such as older age, partner preference, and higher socio-economic status, were significant predictors for requesting epidural analgesia [11, 12]. A higher socioeconomic status may not affect our study population because the Saudi government provides free healthcare and patients do not have to pay for epidural analgesia or any other procedures. Additionally, the results of our study regarding partner preference were not consistent with different studies that reported partner preference as influential [13, 14]. This finding was inconsistent with our observations, as most of the partners were neutral toward epidural use. The inconsistency with our finding is attributed to the fact that we evaluated partner preference from the primigravid point of view and did not ask the partners themselves. When we assessed primigravid women’s decisions to request an epidural before they were educated, most of the participants did not prefer or were undecided on the matter. Although they understood that epidural analgesia was one of the best methods to relieve labour pain, they were concerned about the side effects of epidural analgesia as demonstrated in previous studies performed in Saudi Arabia [6–9]. Their decisions and concerns are justifiable given that greater than half of our studied population indicated that family and friends were their main source of information about epidural analgesia followed by the Internet and physicians. Our findings were similar to the study performed in Riyadh that reported that Saudi women typically learn about epidural analgesia from family and friends and that these individuals were their main source of information regarding epidural analgesia [6]. However, in the US, women ranked physicians as their number one source of information before family, friends and even health educators [14]. Family and friends or the Internet are not reliable or evidence-based sources of medical information. Moreover, primigravid women may not receive all the information needed to make informed decisions. Therefore, healthcare professionals should disseminate information regarding epidural analgesia and present themselves as the primary source of information in this matter. Healthcare professionals should explain the risks and benefits of the procedure, especially in the third trimester.

After the educational session about epidural analgesia, we re-assessed the women’s decisions to request epidural analgesia, and more women expressed the desire to request an epidural in the future. Our findings are consistent with those of a study reporting an increased rate of epidural analgesia requests among women who attended parenting classes in Sweden [15]. Furthermore, the reason for requesting an epidural in nulliparous women was being informed about epidural analgesia before labour [12]. In contrast to our study, 61% of women declined epidural analgesia after antenatal counseling in a study of a Latino population [10]. Educational material can be a powerful tool for clarifying the risks of epidurals to the mother; these risks represent the major concern leading to the avoidance of epidural analgesia. Therefore, it was possible to increase the number of requests for an epidural in our population after providing relevant education. In addition, women who agreed to request epidural analgesia preferred to be educated during consultations with their doctor. This finding can be explained by the fact that most education regarding pregnancy in NGHA occurs at the doctor’s office and occasionally with a health educator if pregnant women have a health issue, such as gestational diabetes mellitus, and need to be educated about insulin and diet. In addition, most patients exclusively attend their doctors’ appointments and have developed a relationship with their doctors. Therefore, these women prefer to be educated by their doctors.

### Strength and weaknesses

Numerous studies evaluated women’s awareness about epidural analgesia. To the best of our knowledge, this study is the second performed in Riyadh (2013) and third in the country [6]. Additionally, to the best of our knowledge, this is the first study to implement an antenatal education session in primigravid women in Saudi Arabia. A trained health educator performed data collection. During the survey and education, we limited the time between the education session and survey completion to prevent the influence of any other intervention that may have played a role as a cofactor in our study. Regarding weaknesses, our survey was based on a small sample size of patients in an outpatient OB/GYN clinic in one public hospital in Riyadh and did not cover other practices. Therefore, this study does not represent the entire population of Saudi Arabia. Despite the small sample size, we experienced a longer duration of sample collection. We encountered problems collecting data because of the implementation of a new software system (named as BEST CARE) at the NGHA hospital. The new software prevented us from booking patients with the health educator clinic. In addition, we did not follow-up with primigravid intrapartum to evaluate their attitude and experience with epidural analgesia.

### Study limitations and further research

The study involved a self-administered questionnaire; thus, an increased risk of report bias is possible. [16] Additionally, primigravid women who said they would request epidurals after the education session were not followed up post-partum to ensure that they requested epidurals. The opinion of partners was reported, although partners were not directly involved in the current study. The responses given by the women are only perceptions of partners’ possible responses. These points should be considered for further research concerning this topic.

## Conclusion

This study demonstrated that education on epidural analgesia can influence women’s decisions. Therefore, we recommend implementing an antenatal education program for all primigravid women to educate them about epidural analgesia and help them make informed decisions.

## Additional file

Additional file 1: Questionnaire. (DOCX 397 kb)

## Abbreviations

NGHA: National Guard Health Affairs
OB/GYN: Obstetrics and Gynecology

## References

[1] Nancy K. ScienceDirect, the nature of labor pain (2002) http://www.sciencedirect.com/science/article/pii/S0002937802701798. Accessed 14 Mar 2017.

[2] Anim-Somuah M, Smyth RMD, Jones L. Epidurals for pain relief in labour, Cochrane. 2011. http://www.cochrane.org/CD000331/PREG_epidurals-for-pain-relief-in-labour. Accessed 26 Dec 2017.

[3] Stark M. Exploring Women's Preferences for Labor Epidural Analgesia, the Journal of Prenatal Education. 2003. https://www.ncbi.nlm.nih.gov/pmc/articles/PMC1595147/#citeref9. Accessed Dec 2017. 10.1624/105812403X106793 PMC1595147 17273336

[4] RÄISÄNEN S, KOKKI M, KOKKI H, GISSLER M, KRAMER M, HEINONEN S. The use of epidural analgesia for intrapartum pain relief in publicly funded healthcare, Acta Obstetricia et Gynecologica Scandinavica. 2014. 10.1111/aas.12268. Accessed Dec 2017. 10.1111/aas.12268 24433265

[5] Orange Flavia Augusta de, Passini-Jr Renato, Melo Adriana S.O., Katz Leila, Coutinho Isabela Cristina, Amorim Melania M.R. Combined spinal-epidural anesthesia and non-pharmacological methods of pain relief during normal childbirth and maternal satisfaction: a randomized clinical trial. 2012. http://www.scielo.br/scielo.php?script=sci_arttext&pid=S010442302012000100023&lng=en&nrm=iso&tlng=en. Accessed Mar 2017. 22392325

[6] Hanem F, et al. Women’s Awareness and Attitude toward Epidural Analgesia. Biology and Agriculture and Health Care. 2013. http://www.iiste.org/Journals/index.php/JBAH/article/view/5680. Accessed May 2016.

[7] Kingdom of Saudi Arabia Ministry of National Guard Health Affairs. http://ngha.med.sa/English/MedicalCities/Jeddah/MedDepartments/Pages/ObGyn.aspx. Accessed 24 Dec 2017.

[8] Oladokun A (2009). Awareness and desirability of labor epidural analgesia: a survey of Nigerian women. Int J Obstet Anesth.

[9] Gari A (2017). Awareness of epidural analgesia among pregnant women in Jeddah, Saudi Arabia. Electronic Physician.

[10] Orejuela J, Garcia F, Green T (2012). Exploring factors influencing patient request for epidural analgesia on admission to labor and delivery in a predominantly Latino population. J Immigr Minor Health.

[11] Hueston W (1994). Factors associated with the use of intrapartum epiduralanalgesia. Obstet Gynecol.

[12] Ramorasata JA (2011). Factors justifying the choice of labor epidural analgesia by nulliparous women: experience at a maternity center in Antananarivo, Madagascar. Med Trop (Mars).

[13] Harkins J, Carvalho B, Evers A, Mehta S, Riley ET. Survey of the Factors Associated with a Woman's Choice to Have an Epidural for Labor Analgesia. Anesthiology Research and Practice. 2010. https://www.hindawi.com/journals/arp/2010/356789/. Accessed May 2016. 10.1155/2010/356789 PMC2915618 20721286

[14] Orbach-zinger S, Bardin R, Berestizhevsky Y, et al. A survey of attitudes of expectant first-time fathers and mothers toward epidural analgesia for labor. International Journal of Obstetric Anesthesia. 2008. http://www.obstetanesthesia.com/article/S0959-289X(08)00013-7/pdf. Accessed Dec 2017. 10.1016/j.ijoa.2008.01.012 18499434

[15] Fabian HM, Rådestad IJ, Waldenström U. Childbirth and parenthood education classes in Sweden. Women's opinion and possible outcomes. Acta Obstetricia et Gynecologica Scandinavica. 2005. http://onlinelibrary.wiley.com/doi/10.1111/j.0001-6349.2005.00732.x/abstract. Accessed Dec 2017. 10.1111/j.0001-6349.2005.00732.x 15842207

[16] Althubaiti A (2016). Information bias in health research: definition, pitfalls, and adjustment methods. J Multidiscip Healthc.

