# Supplementary material for: The effect of epidural education on Primigravid Women’s decision to request epidural analgesia: a cross-sectional study
Source: BMC Pregnancy Childbirth. 2018 May 3;18:124. doi: 10.1186/s12884-018-1766-5 (PMC5934814; doi:10.1186/s12884-018-1766-5)

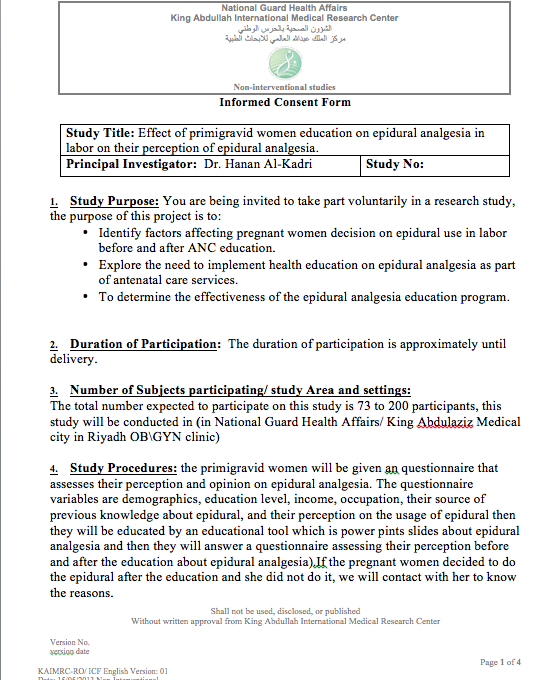

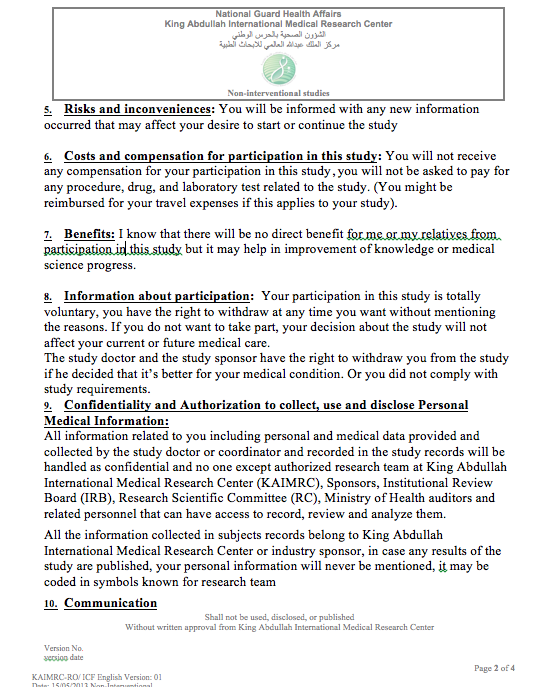


Study date:

Study code:

Study number:

Serial number:

Age: ________yrs.

**General information:**

1. **How much schooling have you completed?**

- Primary school
- Middle School
- High School
- College
- Postgraduate
- Illiterate

1. **What is your** employment status **?**
   - Employed
   - Not employed
2. **Can you give a rough estimate of the family income per month?**

- Less than 5,000 SR
- 5,000 to ≥10, 000 SR
- 10,000 to ≥15, 000 SR
- More than 15,000 SR

1. **Where is your residency?**
   - Urban
   - Rural

**Question related to the research:**

1. **What do you think is the most effective means of pain control in labour?**

- No effective method
- Pain relief with intramuscular analgesia
- Pain relief with intravenous analgesia
- Pain relief by using gas inhalation
- Epidural analgesia
- Others……
- Don’t know

1. **Before coming to the hospital, what was the number one reason why you might have wanted an epidural for labour? (Check only one item)**

| - Pain control |
| --- |
| - Relief of fatigue/stress |
| - Encouraged to obtain epidural by friend/family member |
| - Encouraged to obtain epidural by OB, midwife, labour educator. |
| - Other, please list___________. |
|  |

1. **Before coming to the hospital, what was the number one concern you had regarding epidurals, which may have led you towards avoiding an epidural for labour? (Check only one item)**

| - Concern over possible risks to me (back pain, headache, etc.) |
| --- |
| - Concern over possible risks to baby |
| - Pain from needle/procedure |
| - Afraid of delaying labour or increasing the risk of C-section |
| - Cost |
| - Desire for natural childbirth |
| - Discouraged by OB, midwife, doula, labour educator |
| - Other, please list_____________ . |

1. **What was your main source of information on epidurals prior to your labour? (Check only one item)**

| - Physician |
| --- |
| - Midwife |
| - Family member/friend |
| - Book/video/TV program |
| - Childbirth class |
| - Internet |
| - Other, please list______________.  1. **Did your partner prefer you receive an epidural for labour?**  - Yes - No - Neutral (He thinks this is your own decision to make) |

1. **In the future, will you request an epidural when in labour?**
   - Yes
   - No
   - Not decided

**Answer the following question after receiving education about epidural:**

1. **After receiving the education about epidural, will you request an epidural when in labour?**
   - Yes
   - No
   - Not decided
2. **If you plan to request epidural analgesia when in labour, would you want the issue to be introduced to you formally during your antenatal visits?**

- Yes
  - If Yes:
    - By means of a pamphlet that I can read
    - By means of a video that I can watch
    - During antenatal talks by the health educator
    - During the doctors’ consultation
    - In a special session by the anesthetist
- No introduction needed – I understand already


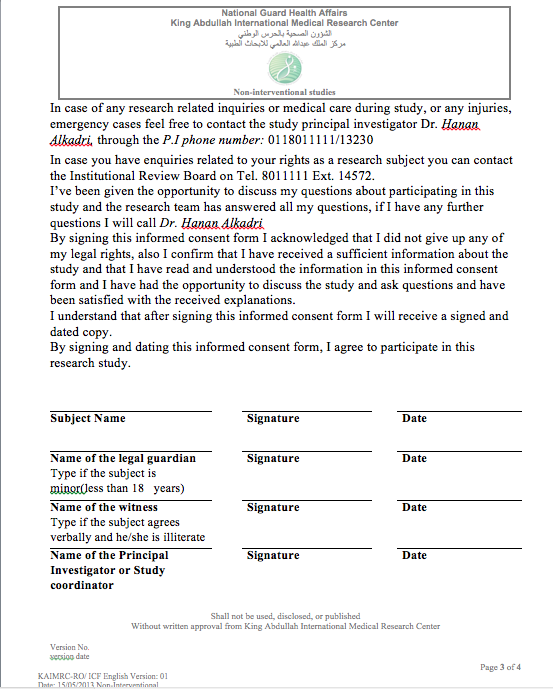

Supplement: Supplementary file 1 — Questionnaire. (DOCX 397 kb) [file 12884_2018_1766_MOESM1_ESM.docx]
